# Mosaic and partial monosomy of chromosome 21 in a case with low platelets count

**Published:** 2014-03-15

**Authors:** A Hashemi, MH Sheikhha, MA Manouchehri, SM Kalantar

**Affiliations:** 1 Department of Pediatric Hematology oncology and Genetic Research Center, Shahid Sadoughi University of Medical Sciences and Health Services, Yazd, Iran; 2 Associate Professor in Genetics, PhD, Genetic Dept. Research & Clinical Centre for Infertility, Yazd, Iran; 3 Pediatric, Main University Hospital, Yazd Medical Sciences University, Yazd-Iran; 4 Associate Professor in Genetics, PhD, Genetic Dept. Research & Clinical Centre for Infertility, Yazd, Iran

**Keywords:** Congenital anomalies, Chromosome 21, Partial monosomy, Platelets

## Abstract

**Background:**

Monosomy is defined as the presence of only one chromosome instead of two in humans. Partial monosomy occurs when only a portion of the chromosome is present in a single copy, while the rest has two copies. It can occur in unbalanced translocations or deletions.

**Case report:**

In this report, a 6 years old girl was presented who was referred to the Pediatric Dep, Shahid Sadoughi Hospital,Yazd, Iran, due to multiple congenital anomalies such as: frontal bossing, horizontal palpebral fissure, small deepest eyes, aplastic nasal bridge, broad philtrum, low set ears, large prominent ears, short neck, microcephaly, pectus excavatum, mental retardation, and dislocation of the hip.

In peripheral blood smear, platelets were decreased but other hematological levels were normal. The karyotype result indicated a mosaic monosomy and partial monosomy of chromosome 21.

**Conclusion:**

According to this and other case reports of monosomy of chromosome 21, this disease had very low prevalence rate among live infants or children. The present case had some congenital anomalies that present with abnormal medical condition. Therefore these patients must be evaluated for chromosomal studies.

## Introduction

Monosomy 21 as a sole cytogenetic abnormality has been reported in a wide variety of hematological disorders, a clear clinical pattern has yet to emerge. Until now, 47 cases of monosomy 21 mosaicism have been described in the literature (1). In this report, a 6 years old girl was presented who was referred to the Pediatric Dep, Shahid Sadoughi Hospital, Yazd, Iran, with partial monosomy of chromosome 21.

## Case report

The case is a 6 years old female that was referred to the Pediatric Dept. due to multiple congenital anomalies. She was the first daughter of non-relative parents who was born via normal vaginal delivery. 

On physical examination, the case had multiple congenital anomalies such as: frontal bossing, horizontal palpebral fissure, small deepest eyes, aplastic nasal bridge, broad philtrum, low set ears, large prominent ears, short neck, microcephaly, pectus excavatum, mental retardation, and dislocation of the hip. In physical examination, there was no lymph adenopathy or thyromegally. The spleen was palpable but there was no hepatomegaly. The lung and heart sounds were normal. 

The case had a history of epilepsy at age of 9 months which had been controlled by phenobarbital and nitrazepam tablets. In addition, there was a history of urinary infection with normal findings in sonography. 

In peripheral blood smear, platlates were decreased but other hematological levels were normal. The cases laboratory data is presented in table 1.

In bone morrow aspiration she had normal cellularity and the megakaryocytic level was increased and myeloid level was seen until neutrophils production. There was no malignant cell in the blood sample. According to the results of bone morrow aspiration and her clinical condition, 3 times platelets transfusion was done for her. 

For genetic evolution karyotype was performed. To do this, fifty metaphase spreads were studied on the basis of GTG technique at 450 band resolution. In 11 spread monosomy of chromosome 21 was detected, while in the remaining 39 spreads, 46 chromosomes with marker chromosomes were observed. The marker chromosome is most probably a chromosome 21 with deletion at band distal to q21 (21q21). Therefore the karyotype result indicated a mosaic monosomy and partial monosomy of chromosome 21 (45, XX, - 21 [11] / 46, XX, del (21)(q21) [39]). The cytogenetic test was performed for the parents and it was normal. In view of her parent's karyotype this chromosomal aberration in the case is of de novo origin, and the mosaic pattern is of post–zygotic origin. It was explained to the parents that the risk of recurrence in future pregnancies is around 1%. 

## Discussion

In this study, a 6 years old girl with monosomy of chromosome 21 and many congenital anomalies that were presented in her phenotype was reported. By cytogenetic study, she had mosaic monosomy and partial monosomy of chromosome 21. There were some case reports in the literature that have at least some similarities with our case. Chettouh, et al (1995) compared the phenotypes, karyotypes, and molecular data for six cases of partial monosomy 21. In their report 5 regions of chromosome 21 that the deletion of which corresponds to particular features of monosomy 21, were defined.(2)

Chang, et al (1995) reported 50 cases of acute nonlymphocytic leukemia (ANLL). Their cytogenetic data showed that 2 patients carry a monosomy 21 abnormality which has been rarely reported in hematologic malignancies. The first case was a 58-year-old male with the diagnosis of AML, FAB M2, who died of refractory leukemia 9 months later. The other case was a 59-year-old female with AML, FAB M2. Complete remission was achieved initially but she died of sepsis 3 months later with no evidence of leukemic relapse (3). Monosomy 21 is not yet recognized as a nonrandom cytogenetic abnormality in ANLL, whereas its unusual predilection in AML, especially the FAB M2 or M4 categories, as noted in the above study, have raised this possibility (3). A patient with full monosomy 21 detected from routine GTG-banded karyotyping was reported by Riegel et al (2005). An unbalanced translocation between the long arms of chromosomes 18 and 21 was found in re-examination of this case with chromosome painting demonstrated (4). Flaherty, et al (1998) presented a case with monosomy 21 that was detected by routine cytogenetics. The fluorescence in situ hybridization (FISH) study demonstrated an unbalanced translocation t(5;21). The patient was partially monosomic for both 5p and 21q (5). Plomp, et al (1998) reported two mentally retarded adults with an unbalanced karyotype resulting from a familial balanced translocation between chromosomes 8 and 21. Both patients had partial trisomy 8p and partial monosomy 21q (6). Orti, et al (1997) reported that deletion of genes from the chromosome 21 region between APP and SOD1 is a potential cause of some of the major phenotypic features of monosomy 21 patients. Furthermore, FISH mapping of two patients with partial monosomy 21 using YAC and cosmid clones defined more accurately the telomeric border of the critical region between markers S226 and S213 (7). 

## Conclusion

According to other related case reports of monosomy of chromosome 21, this disease had very low prevalence rate among live infants or children. The present case had some congenital anomalies that present with abnormal medical condition. 

These patients must be evaluated for detecting facial phenotype and other signs that discussed in medical research books. 

## Conflict of interest

The authors have no conflict of interest.
